# Assessment of Peptides and Membrane Physico-Chemical Characteristics on Migration Selectivity and Recovery of Antimicrobial Fractions Using Electrodialysis with Ultrafiltration Membrane on a Calf Cruor Hydrolysate

**DOI:** 10.3390/membranes16060202

**Published:** 2026-06-10

**Authors:** Véronique Perreault, Jacinthe Thibodeau, Sara García-Vela, Laurent Bazinet

**Affiliations:** Food Science Department, Université Laval, Institute of Nutrition and Functional Foods (INAF), Laboratoire de Transformation Alimentaire et Procédés ÉlectroMembranaires (LTAPEM/Laboratory of Food Processing and ElectroMembrane Processes), Centre de Recherche Sur Les Protéines (PROTEO-ULaval), Quebec Network for Research on Protein Function, Engineering and Applications (PROTEO), Dairy Science and Technology Research Centre (STELA), Québec, QC G1V 0A6, Canada; veronique.perreault.5@ulaval.ca (V.P.); jacinthe.thibodeau.1@ulaval.ca (J.T.); sara.garcia-vela.1@ulaval.ca (S.G.-V.)

**Keywords:** calf cruor, hydrolysate, electrodialysis with ultrafiltration membranes, peptide selectivity, peptide physico-chemical characteristics, antimicrobial activity

## Abstract

In recent years, cruor from slaughterhouse blood has garnered growing interest as a potential source of antimicrobial peptides obtained through enzymatic hydrolysis. In addition, electrodialysis with ultrafiltration membrane (EDUF) represents a strategy for valorizing peptide-rich hydrolysates, enabling the selective separation and concentration of antimicrobial peptides, according to their size and charge. Hence, this study evaluated the potential of EDUF to fractionate, for the first time, calf cruor hydrolysate and explore its use as a novel source of antimicrobial peptides. The resulting peptide fractions were characterized to investigate the selectivity of peptide migration in relation to peptide physico-chemical characteristics and membrane properties and to finally assess their antimicrobial activity. High migration rates of 12.75 ± 2.17 g/m^2^h and 8.94 ± 0.38 g/m^2^h were observed for the cationic (P^+^) and anionic (P^−^) recovery fractions, respectively. These results suggested that peptide migration from calf cruor hydrolysate to both recovery fractions during EDUF was influenced by the combined effects of molecular weight, net charge, hydrophobicity, specific amino acid residues (L, Y), and peptide–membrane interactions. Furthermore, the initial and final hydrolysates as well as P^+^ fractions exhibited antifungal activities against *Paecilomyces* spp. and *Rhodotorula mucilaginosa* with minimum inhibitory concentrations (MIC) ranging from 0.312 to 0.615 mg/mL and minimum fungicidal concentrations (MFCs) ranging from 0.312 to 1.250 mg/mL. In contrast, the P^−^ fraction did not exhibit antifungal activity, but a slight anti-*Listeria* activity was detected, with a MIC of 10 mg/mL. These findings highlight the potential of upcycling calf blood into functional antifungal and antibacterial agents, supporting a circular economy approach and transforming waste streams into value-added ingredients that enhance food preservation.

## 1. Introduction

In recent years, increased global pressure has been exerted on the food industry to minimize its environmental impact and enhance its sustainability. This has driven growing interest in the comprehensive recovery and optimal utilization of food industry by-products [1,2]. Among these, blood is produced in substantial quantities as a by-product in slaughterhouses worldwide. While its production poses a significant environmental concern due to its high pollutant load, it also offers considerable potential for valorization due to its protein-rich content. Cruor, the solid fraction of blood obtained after centrifugation, accounts for around 40% of the total blood volume and contains 70–75% of the overall blood protein content [3]. Composed of more than 90% hemoglobin, cruor is mainly responsible for blood’s unstable red coloration. Cruor serves as an ideal substrate for enzymatic hydrolysis, enabling the production of bioactive peptides [4,5] which are part of a sustainable strategy to reduce industrial waste and generate high-value products [2,6,7]. Indeed, antimicrobial activities were reported for several sources of cruor hydrolysates such as pork, bovine, chicken, turkey, and calf [3,5,8,9,10,11]. Therefore, peptides sourced from hemoglobin represent promising natural alternatives to synthetic antimicrobial agents for use in food preservation [12,13]. However, hemoglobin coming from these different cruor sources possess distinct amino acid sequences, and the variability in hydrolysis conditions such as pH, duration, and enzyme used significantly influences the composition of the resulting peptide populations as well as their bioactivities [10,11,14]. Peptide antimicrobial activities are influenced by several structural factors, including their amino acid sequence, molecular weight, terminal amino acid type and charge, and three-dimensional conformation, as well as their hydrophilic and hydrophobic characteristics [6,15].

Although antimicrobial peptides have previously been identified in calf cruor [9], the efficient enrichment of these bioactive components from this complex hydrolysate mixture remains difficult. The hydrolysates generated by enzymatic hydrolysis are usually submitted to fractionation and purification processes to produce enriched peptide fractions to increase their bioactivity and to remove compounds that may interfere with peptide function through agonistic or antagonistic effects [16,17,18]. However, to obtain successful separation of peptides of interest, their physico-chemical characteristics must be considered. Hence, in comparison with other techniques, electrodialysis with ultrafiltration membranes (EDUF) integrates ion exchange and ultrafiltration membranes in an electrically driven system. This combined mechanism enables peptide fractionation according to the molecular weight and it offers a double peptide selectivity since both anionic and cationic species can be separated simultaneously. EDUF can be optimized (electric field, pH, membrane properties, etc.) to selectively recover and enrich target peptides and has been successfully used for the separation and concentration of bioactive peptides [4,19,20]. Hence, EDUF is a promising technology for the valorization of liquid wastes from the food industry, enabling the recovery of valuable compounds such as peptides and supporting the development of circular economy [21].

When subjected to an electric field, peptides migrate toward the anode or cathode based on their net charge, and are separated according to their molecular weight, as determined by the molecular weight cut-off of the filtration membrane [4,22]. The separation of peptides by EDUF has already been performed on blood porcine cruor hydrolysate [4] and pure bovine hemoglobin hydrolysate [23], but to date, no study has reported the separation of peptides from hydrolysates derived from calf cruor. Furthermore, since the calf hemoglobin sequence is different from other sources in terms of amino acid residues [24,25], the hydrolysis of this source will give a different peptide population and after EDUF separation, it will lead to different bioactivities. This represents a novel contribution to the field, considering that the number of calf carcasses processed in 2023 approached 195,000 in Canada only (Agriculture and Agri-Food Canada, 2023) [26]. In addition, given the distinct physico-chemical characteristics of calf cruor hemoglobin [9] and the underutilization of its protein-rich by-products, exploring its potential to produce bioactive peptides is relevant regarding sustainability and circular economy.

Since ultrafiltration membranes are used in the EDUF process, peptide and membrane interactions must also be considered. Indeed, several statistical approaches such as redundancy and multivariate regression analyses or machine learning tools have been previously used to explain how the physico-chemical characteristics of filtration membranes such as porosity, charge, roughness, etc., influence the migration of individual peptides from whey protein hydrolysate and porcine cruor hydrolysate [27,28,29]. Moreover, specific physico-chemical properties of peptides involved in their migration (ex. isoelectric point, molecular weight and net charge of the peptides, specific amino acid residues, etc.) were found to be associated with their affinity for filtration membranes [27,28]. Analyzing peptide and filtration membrane interaction has been performed on whey hydrolysate and porcine cruor hydrolysate [27,28,29], but it has never been studied on calf cruor hydrolysate, a source of peptides with distinct amino acid sequences.

In this context, the aims of the present study were to (1) explore for the first time the separation of a calf cruor hydrolysate by electrodialysis with ultrafiltration membranes, (2) evaluate the process performances of EDUF, (3) characterize the resulting peptide fractions by elucidating the mechanisms governing selective peptide migration based on peptide and membrane physico-chemical properties, and (4) evaluate the antifungal and antibacterial activities of the different peptide fractions recovered after EDUF.

## 2. Materials and Methods

### 2.1. Materials

#### 2.1.1. Calf Cruor

Fresh calf blood was collected from six animals at the Saint-Germain slaughterhouse (Montpak International Inc., Saint-Germain-de-Grantham, QC, Canada). EDTA was added immediately during collection at a concentration of 1 g/L to prevent coagulation. The blood was transported under refrigerated conditions to the laboratory for cruor preparation, according to Sanchez-Reinoso et al. [9].

#### 2.1.2. Chemicals

Pepsin from porcine gastric mucosa (EC 3.4.23.1; 3200–4500 U/mg protein) and ethylenediaminetetraacetic acid tetrasodium salt dihydrate (EDTA) were obtained from Sigma-Aldrich (St. Louis, MO, USA). Hydrochloric acid (HCl), sodium hydroxide (NaOH), and sodium chloride (NaCl) were purchased from Fisher Scientific (Ottawa, ON, Canada). All solvents and reagents used for RP-UPLC-MS/MS analyses were LC-MS grade and supplied by Fisher Scientific (Ottawa, ON, Canada). For antimicrobial activity assays, Tryptic Soy Agar (TSA), natamycin, and ampicillin were purchased from Millipore Sigma Canada Ltd. (Oakville, ON, Canada), Potato Dextrose Agar (PDA), peptone, and Dichloran Rose Bengal Chloramphenicol Agar (DBRC) were from BD Difco Laboratories (Sparks, MD, USA) and Tryptic Soy broth was from BD Bacto™ (Franklin Lakes, NJ, USA).

### 2.2. Production of Calf Cruor Hydrolysates

#### 2.2.1. Calf Cruor Hydrolysis

Hydrolysis was carried out as described by Sanchez-Reinoso et al. [9] with slight modifications. Calf cruor containing 98.7% proteins (dry basis) was hydrated overnight at 4 °C at a concentration of 1% (*w*/*v*) based on protein content. The temperature of the mixture was raised at 37 °C, and the pH was adjusted to 3.0 using 3 M HCl, since the best antimicrobial activities were reported at this pH [9,10,11]. The solution was left at pH 3.0 for 15 min to allow hemoglobin denaturation. Pepsin was added at an enzyme-to-substrate ratio of 1/11 mol/mol and hydrolysis was performed for 30 min while keeping the pH, temperature, and agitation constant. Samples were taken at 2.5, 10, 15, 20, and 30 min of hydrolysis. The reaction was stopped by increasing the pH to 10.0 with 5 M NaOH and holding for at least 30 min. After 30 min, a degree of hydrolysis [30] of 7.6 ± 0.2% was obtained and confirmed a cleavage pattern indicative of a zipper-like mechanism, in accordance with the results reported in the study of Sanchez-Reinoso et al. [9].

#### 2.2.2. Discoloration of Calf Cruor Hydrolysate

Discoloration of final calf cruor hydrolysate (CCH) was performed, to discard the heme from hemoglobin and avoid potential fouling on the membrane that would limit the efficiency of EDUF, by lowering the pH to 4.0 with 5 M HCl, followed by a subsequent decantation at 4 °C for 24 h. The solution was then centrifuged at 6000× *g* for 30 min at 23 °C. The supernatant consisting of the discolored hydrolysate was adjusted to pH 7.0 with 1 M NaOH and was freeze-dried before use for EDUF separation [8].

### 2.3. Separation of Calf Cruor Peptides by EDUF

#### 2.3.1. Configuration

**Figure 1:**
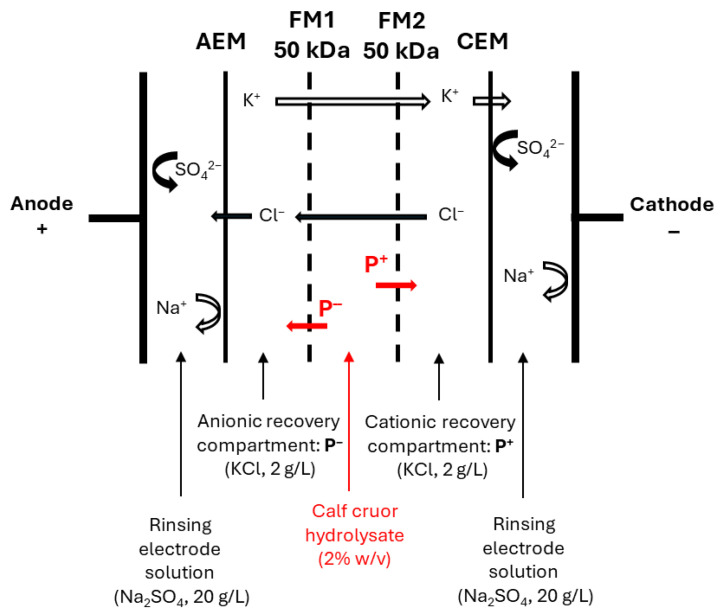
Electrodialysis with ultrafiltration membrane configuration for the simultaneous recovery of anionic and cationic peptides. AEM: anion exchange membrane; FM: filtration membrane; CEM: cation exchange membrane; P^−^: negatively charged peptides; P^+^: positively charged peptides.

An electrodialysis (ED) stack using the MP type cell (Electrocell Systems AB, Täby, Sweden) featuring an effective membrane area of 100 cm^2^ was employed. The configuration included two electrodes, a stainless-steel cathode and a dimensionally stable anode (DSA) composed of titanium, both sourced from Electrocell Systems AB. The ED cell assembly consisted of two polyethersulfone (PES) filtration membranes (50 kDa molecular weight cut-off (MWCO), Synder Filtration, Vacaville, CA, USA) positioned between food-grade ion exchange membranes, an anion exchange membrane (AEM/AMX-fg), and a cation exchange membrane (CEM/CMX-fg), supplied by Astom (Tokyo, Japan) (Figure 1). This filtration membrane with a MWCO of 50 kDa was selected since, as reported in the literature, it allows for higher peptide migration rates in the recovery fractions. In addition, this membrane was also selected since the selectivity of the peptides was not affected compared to lower MWCO membranes and it was less prone to fouling [4,28,31]. In addition, since the electric field is the only driving force for the EDUF process [32], larger pores are necessary, even to migrate low molecular weight peptides.

This configuration was organized into five distinct chambers creating four closed-loop circuits, each linked to external reservoirs enabling continuous solution recirculation. The electrode rinse loop contained 1000 mL of sodium sulfate (Na_2_SO_4_) solution at a concentration of 20 g/L, recirculated at a flow rate of 800 mL/min. The circuits assigned for anionic (P^−^) and cationic (P^+^) peptide fraction collection were each filled with 600 mL of potassium chloride (KCl, 2 g/L) and maintained at a flow rate of 700 mL/min. The central feed loop consisted of 600 mL of a 2% (*w*/*v*) discolored hydrolysate solution derived from calf cruor, adjusted to pH 7.0 and circulated at 700 mL/min.

#### 2.3.2. Protocol

Preliminary tests were conducted to determine the limiting current density (LCD), using the approach described by Cowan and Brown [33]. The current intensity was monitored by voltage increments of 0.5 V, from 0 to 30 V. The resistance was plotted (ratio of voltage/intensity as a function of the reciprocal current) and the intersection of the extrapolated tangential lines of the ohmic plateau sections allowed for the determination of the limiting current density corresponding to the appearance of water splitting phenomenon [34]. Based on the specific cell configuration (Figure 1) and operational conditions described in Section 2.3.1, the LCD was estimated at 301 A/m^2^, corresponding to a voltage of 19 V and a current of 3.01 A. Following the LCD determination, the applied voltage for subsequent experiments was set at 7.8 V, resulting in an electric field strength of 2 V/cm between electrodes (distance of 3.9 cm between the electrodes), chosen for consistency and to compare the results with previously published protocols [19,20,28].

Voltage was monitored in real time using a multimeter connected directly to the electrode terminals, with power supplied by a 0–30 V DC source (HQ Power PS3003, Xantrex, Burnaby, BC, Canada). All flow rates across the circuits were maintained constant throughout the EDUF process. Initially, the conductivity of the KCl containing recovery solutions (P^−^ and P^+^) was matched to that of the feed solution and allowed to gradually increase during treatment, ensuring a continuous and controlled migration of charged peptide species [19].

The pH of CCH and recovery solutions was adjusted to 7.0 prior to the run to ensure the simultaneous separation of both anionic and cationic peptides and was kept stable throughout via the addition of 0.1 N NaOH or HCl solutions as needed. Operational parameters, including conductivity, pH, and amps, were measured at 15 min intervals during the process. Each EDUF run lasted 180 min, with samples collected prior to voltage application and, subsequently, at 30 min intervals. Upon completion, the separated peptide fractions from each compartment were freeze-dried and stored at −20 °C for later analysis. All experiments were independently replicated three times, with fresh membrane sets used for each trial to ensure experimental reproducibility.

### 2.4. Analyses

#### 2.4.1. Protein/Peptide Content

The total nitrogen content of the discolored hydrolysate before and after EDUF and the recovery compartment solutions P^−^ and P^+^ were determined on freeze-dried samples using a rapid MicroNcube (Elementar, Langenselbold, Germany), based on the Dumas method. The protein concentration was estimated with a conversion factor of 6.25 [35].

#### 2.4.2. EDUF Process Analyses

*pH and Conductivity*

In each compartment, pH was monitored using a pH meter (Model Star A221 Portable pH-meter, Thermo Scientific, MA, USA) while conductivity was monitored using an YSI conductivity meter (Model 3100 Yellow Springs, OH, USA).

*Mass Transfer: Global Peptide Migration Rate in Recovery Fractions*

The concentration of peptides in the liquid fractions collected from the P^−^ and P^+^ recovery fractions was quantified using the micro-BCA (µBCA) protein assay kit (Thermo Fisher Scientific, Waltham, MA, USA). A calibration curve was generated using bovine serum albumin (BSA) as the standard protein. For the assay, 150 µL of either the BSA standards or EDUF-derived samples (diluted 20-fold) were pipetted into a microplate, followed by the addition of 150 µL of working reagent. The microplate was then incubated for 2 h at 37 °C. Absorbance measurements were taken at 562 nm using an xMark microplate spectrophotometer (Bio-Rad, Mississauga, ON, Canada) and peptide concentrations were calculated based on the standard curve, expressed in µg/mL.

To evaluate the mass transfer efficiency, the flux of migration or global migration rate (MR) was calculated. This was based on the total amount of peptides (µg) quantified in each recovery compartment, multiplied by the volume recovered, and normalized over the total treatment time. The migration rate was calculated using Equation (1):

(1) $$MR=\frac{m}{s\;\times t}$$

where *MR* is expressed in g/m^2^h, *m* the mass of peptides in gram (g), *s* the filtration membrane surface (0.01 m^2^), and *t* is the duration in hours (h) of the separation process.

*Global System Resistance*

The overall electrical resistance of the system (*R*, in Ω) was calculated using Ohm’s law (Equation (2)):

(2) $$R=\frac{U}{I}$$

Voltage (*U*, in V) and current (*I*, in A) were continuously recorded from the power supply to monitor system performance and allow resistance calculation throughout the process.

*Relative Energy Consumption*

The relative energy consumption (REC) was calculated using Equation (3), adapted from Deboli et al. [36]:

(3) $$REC=\;\frac{\int_{0}^{t}U\;\times \;I\;\times t\left(dt\right)}{m\;\times \;3600}$$

where *REC* is expressed in Wh/g of peptides, *U* the voltage in volt (V), *I* the current intensity in amp (A), *t* the duration in second (s), and *m* the quantity of peptides in grams (g).

*Membrane Thickness and Electrical Conductivity*

The thickness and electrical conductance of each membrane were both evaluated before and after the EDUF process to assess their conductivity and identify any potential alterations in membrane integrity. Membrane thickness was measured using a digital micrometer (Marathon Watch Company LTD, Richmond Hill, ON, Canada). Electrical conductance was measured using an YSI Model 3100 conductivity meter connected to a custom-designed measurement clip provided by the Institut de Chimie et des Matériaux Paris-Est (ICMPE), Université Paris-Est (Thiais, Val-de-Marne, France). Prior to testing, membranes were conditioned by immersion in a 0.5 M NaCl solution for 30 min. Following the measurements, membrane conductivity was calculated according to Equation (4) [37]:

(4) $$\kappa =\frac{l}{{A\;\times \;R}_{m}}$$

where *κ* is the membrane conductivity (mS/cm), *l* the membrane thickness (cm), *A* the electrode area (1 cm^2^), and *Rm* the membrane resistance (Ω) determined using Equation (5) [38]:

(5) $$R_{m}=\;\frac{1}{G_{m}}=\frac{1}{G_{m+S}}-\frac{1}{G_{s}}=R_{m+s\;}-R_{s}$$

where *G* is the conductance (S), *m* the membrane, *m + s* the membrane in the reference solution, and *s* the reference solution.

#### 2.4.3. RP-UPLC and Mass Spectrometry Analyses

Before conducting the RP-UPLC, mass spectrometry analyses, and antimicrobial assays, all EDUF fractions, including the initial and final hydrolysates as well as the P^+^ and P^−^ recovery fractions, were subjected to a demineralization step using a conventional electrodialysis process to reduce their salt content [39].

The different UPLC-MS/MS analyses were performed in the same conditions and using the same equipment described in Sanchez-Reinoso et al. [9]. Briefly, a 1290 Infinity II UPLC (Agilent Technologies, Santa Clara, CA, USA) was used to perform RP-UPLC analyses. Demineralized initial hydrolysate (ICCH), final hydrolysate (FCCH), and the P^+^ and P^−^ fractions were filtered through 0.45 µm PVDF filters and collected in glass vials. Samples (1 µL, 1% protein concentration determined by the Dumas method) were injected onto a Poroshell 120 EC-C18 column (2.1 × 100 mm, 2.7 µm, Agilent, Santa Clara, CA, USA) for peptide sequence identification, migration selectivity assessment, and statistical analyses.

The mass spectrometer used to identify and quantify the relative abundances of the calf cruor peptides was a hybrid ion mobility quadrupole time-of-flight (6560 IM-Q-TOF, Agilent, Santa Clara, CA, USA) in the conditions described by Sanchez-Reinoso et al. [9] and Cournoyer et al. [8].

#### 2.4.4. Antimicrobial Assays

*Strain Collection and Maintenance*

The antimicrobial activity of the ICCH, FCCH, P^+^, and P^−^ fractions was evaluated against the following indicator microorganisms: the Gram-negative *Escherichia coli* MP 4100, the Gram-positive *Listeria ivanovii* HP B28, the yeast *Rhodotorula mucilaginosa* 27,173, and the mold *Paecilomyces* spp. 5332-9a. *E. coli* was selected since it is a Gram-negative common foodborne pathogen, whereas *L. ivanovii* was chosen as a Gram-positive strain to provide insights into the prevention of listeriosis, a significant concern in certain food products [40]. The mold (*Paecilomyces* spp.) and the yeast (*R. mucilaginosa*) were chosen since they are commonly associated with food spoilage [41,42].

Tryptic Soy Agar (TSA) was used for the growth and maintenance of bacterial isolates, while Potato Dextrose Agar (PDA) was used for fungal isolates. For media preparation, manufacturer instructions were followed and 1% agar was added to Tryptic Soy and Potato Dextrose culture media. Incubation conditions were as follows: 37 °C for 24 h for bacterial strains, 25 °C for 48 h for yeast strains, and 25 °C for 4–5 days for molds, all under aerobic conditions.

*Evaluation of the Antimicrobial Activity*

The agar well diffusion method and microtitration methods were used to evaluate the antimicrobial activities of the EDUF fractions [43]. The protein content from demineralized ICCH and FCCH and P^+^ and P^−^ fractions were adjusted to 40 mg/mL by diluting in Milli-Q water. All assays were performed in triplicate.

Agar well diffusion

ICCH, FCCH, P^+^, and P^−^ fractions were tested using the agar well diffusion method [44] against the indicator strains *E. coli*, *L. ivanovii*, *R. mucilaginosa*, and *Paecilomyces* spp. To perform this procedure, 25 µL of the prepared culture suspension (adjusted to 10^6^ CFU/mL for bacteria and yeasts and 10^5^ CFU/mL for molds) was diluted in 25 mL of Tryptic Soy Broth (TSB) (for bacterial strains) or Potato Dextrose Broth (PDB) (for fungal strains) supplemented with 0.75% agar and poured into a Petri dish (90 mm diameter) until dried. The culture media of the suspension was prepared using TSB for bacterial cultures and peptone water (10% *w*/*v*). Subsequent to the drying process, wells were formed using a sterile 5 mL pipette. A volume of 80 µL of each fraction at a concentration of 40 mg/mL was added to each well. Ampicillin, at a concentration of 256 µg/mL, and natamycin, at 50 µg/mL, were used as positive controls for bacterial and fungal tests, respectively, as they are wide-spectrum antimicrobials. Milli-Q water was included as a negative control in all experiments. Finally, the agar plates were incubated at 37 °C for 24 h for bacterial tests and at 25 °C for 48 h for fungal tests. Thereafter, inhibition halos were measured and images were captured using a Chemidoc camera (Bio-Rad, Mississauga, ON, Canada).

Minimum inhibitory concentration (MIC) and minimum bactericidal/fungicidal concentration (MBC/MFC)

A microtitration assay was conducted to determine the minimum inhibitory concentration (MIC) of the four fractions (ICCH, FCCH, P^+^, and P^−^) against the collection of indicator microorganisms [45]. The growth medium used for the isolated bacterial and fungal strains was TSB and PDB, respectively. In brief, 175 µL of the culture medium was added to the wells of column 1 (negative growth control) and 125 µL to the wells of columns 2–12. Subsequently, 125 µL of each hydrolysate fraction (stock concentration of 40 mg/mL), positive control (stock concentration of 256 µg/mL for ampicillin in bacterial tests and 50 µg/mL for natamycin in fungal tests), and negative control (Milli-Q water) were added to the wells in column 3 and mixed by pipetting up and down 10 times. A total of 125 µL from column 3 was removed and transferred to column 4, where serial dilutions were performed until the final dilution reached column 12. The final volume was then discarded. Thus, the fraction concentration in each well starts at 20 mg/mL and decreases in 1:2. Afterward, 50 µL of the indicator strain suspension (at a concentration of 10^5^ CFU/mL for yeasts and bacteria and 10^4^ CFU/mL for molds) was added to all the wells, except for column 1. The culture medium used for culture suspension was identical to that employed in the agar well diffusion method. The microplate was incubated 24 h at 37 °C for bacterial isolates and for 48 h at 25 °C for fungal isolates. Following the incubation period, the number of wells exhibiting inhibition was recorded by measuring the optical density at 595 nm using a PowerWave XS2 microplate reader (BioTek Instruments, Winooski, VT, USA) and the program Gen5 2.09 [43].

The minimal bactericidal/fungicidal concentration (MBC/MFC) was calculated by transferring 10 µL of the wells where no growth could be detected to agar plates (TSA for bacterial isolates and Dichloran Rose Bengal Chloramphenicol Agar (DBRC) for fungal strains). After incubation (24 h, 37 °C for bacteria and 48 h, 25 °C for fungi), the fraction concentration in which no growth was observed was the MBC/MFC [43]. The MFC/MIC ratio was calculated to ascertain the effect of the peptide fractions [46]. A ratio ≤4 is indicative of bactericidal/fungicidal efficacy, whereas a ratio >4 indicates bacteriostatic/fungistatic activity.

#### 2.4.5. Data Treatment and Statistical Analyses

*UPLC-MS/MS Data Treatment*

The four EDUF fractions (ICCH, FCCH, P^+^, and P^−^) were subjected to statistical treatment using Profinder V10.0.2 and Mass Profiler Professional (MPP) B.15.1 (Agilent Technologies, Santa Clara, CA, USA) according to Cournoyer et al. [4] with some modifications. Profinder allowed for the preparation of the data by extracting compounds according to different parameters (peak heights above 2000 counts and present in at least 2 repetitions in at least one sample group). Then, using MPP, a z-transformation was applied to the dataset and log 2 normalization so peptide ion abundance values in each sample were baselined to the mean of each compound in all samples. Afterward, statistics and interpretations were performed on this filtered dataset: non-parametric Kruskal–Wallis with *p*-value computation (10,000 permutations), *p* ˂ 0.05, and Benjamini–Hochberg FDR multiple testing correction [4]. These statistic parameters allowed researchers to identify which peptides have some differences between conditions. Finally, a hierarchical clustered heatmap on both entities and conditions (setting similarity measure with Euclidean distance metric and Ward’s linkage rule) was generated to examine potential groups of peptides and to study the general behavior of the conditions.

*Other Statistical Analyses*

All EDUF experiments were conducted in triplicate under identical conditions to ensure reproducibility. Accordingly, all results were presented as mean values ± standard deviation. To assess the variation in EDUF parameters during the process (conductivity, pH, global system resistance, peptide migration), regression tests were applied. For the characterization of membranes before and after the EDUF process, a paired *t*-test was used (*p* < 0.05). Statistical analyses were performed with SigmaPlot software (Version 14.0, Systat Software, Inc., San Jose, CA, USA).

## 3. Results and Discussion

### 3.1. Performance Evaluation of the Electrodialysis Process

#### 3.1.1. Evolution of pH and Conductivity

**Figure 2:**
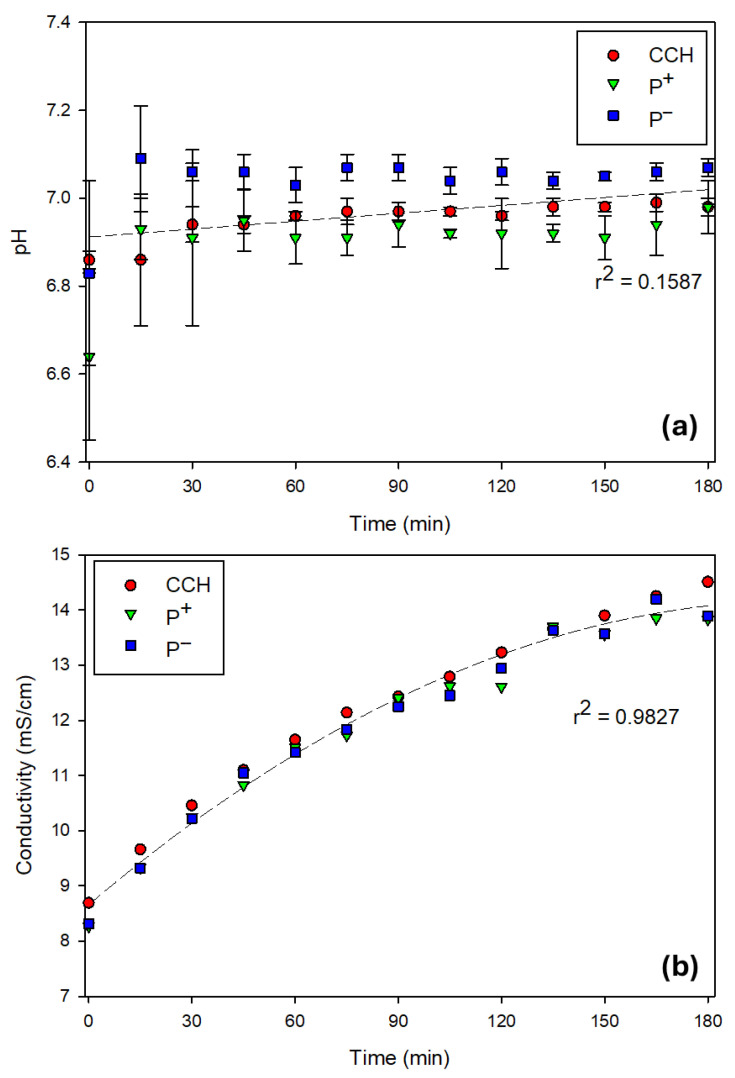
Evolution of (**a**) pH and (**b**) conductivity in the calf cruor hydrolysate and peptide recovery compartment fractions over time during the EDUF process. CCH: calf cruor hydrolysate; P^+^: positively charged peptides; P^−^: negatively charged peptides.

Throughout the EDUF process, pH and conductivity were monitored. The pH was maintained constant at 7.0 with the addition of 0.1 M NaOH or HCl. Hence, for all fractions, the pH evolution remained constant during all process (*p* ˃ 0.05) (Figure 2a). In this study, maintaining the pH at 7.0 was essential to enable the simultaneous separation of anionic and cationic peptides by maintaining their charges throughout the process [47]. According to their isoelectric points (pI), basic peptides (pI > 7) exhibit a net positive charge at pH 7, whereas acidic peptides (pI < 7) exhibit a net negative charge [48]. Consequently, maintaining a constant pH in the peptide recovery solutions P^+^ and P^−^ was necessary, as fluctuations can negatively affect peptide electrophoretic behavior and may result in back migration [4,47,49].

Also, to maintain efficient and continuous peptide separation during the process [50], the conductivity of the recovery compartment fractions was adjusted with KCl powder to match that of the hydrolysate compartment. Therefore, the conductivity of all compartments increased during the separation process (Figure 2b). This increase was significant between the start and the end of the process (*p* < 0.001), but this variation was similar between the three compartments (*p* = 0.921). It started at 8.42 ± 0.23 mS/cm and reached 14.07 ± 0.38 mS/cm at the end of the process. This was in accordance with the EDUF configuration (Figure 1) used since the conductivity of the CCH compartment increased throughout the process due to the migration of K^+^ and Cl^−^ ions. This was accompanied by a depletion of ions in the recovery compartment fractions [51], needing the addition of KCl to maintain the conductivity.

#### 3.1.2. Evolution of Peptide Migration

**Figure 3:**
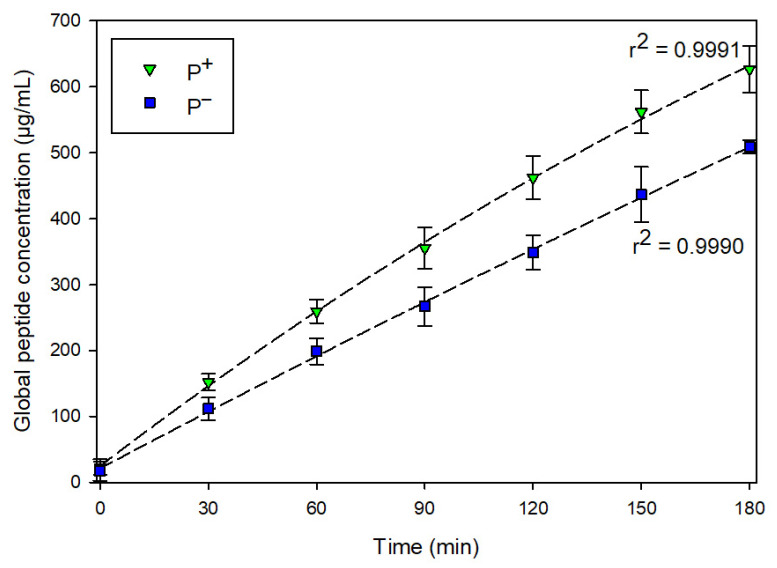
Evolution of the global peptide concentration (µg/mL) in the recovery compartment fractions over time during the EDUF process. P^+^: positively charged peptides; P^−^: negatively charged peptides.

The overall peptide concentration increased quite linearly over time, confirming the migration of positively and negatively charged peptides toward the electrodes (Figure 3). Concentrations of 627 ± 36 µg/mL and 509 ± 10 µg/mL were respectively achieved in the P^+^ and P^−^ compartments. The migration rate was statistically higher in the P^+^ fraction, containing positively charged peptides (*p* = 0.002) with migration rates of 12.75 ± 2.17 g/m^2^h and 8.94 ± 0.38 g/m^2^h obtained for the P^+^ and P^−^ fractions, respectively. The absence of a plateau in peptide migration in both the P^+^ and P^−^ compartments implies that steady-state conditions had not been achieved. Therefore, extending the EDUF duration may increase overall peptide yield in both recovery fractions. These migration rates are higher than those reported by Cournoyer et al. [4] and Vanhoute et al. [23]. Indeed, using a discolored porcine cruor hydrolysate, Cournoyer et al. [4] obtained a cationic peptide migration rate of 5.75 g/m^2^h under a configuration designed exclusively for cationic peptide separation, using the same ultrafiltration membrane (50 kDa PES) and an applied voltage of 5 V. Vanhoute et al. [23], on the other hand, employed the same configuration as the present study with a discolored bovine hemoglobin hydrolysate at pH 7.0 under a voltage of 8 V and obtained a migration rate for the cationic fraction of 5.06 g/m^2^h using 20 kDa cellulose ester filtration membranes. In both cases, the conductivity of the recovery solutions was lower: 7 mS/cm for Cournoyer et al. [4] and 3 mS/cm for Vanhoute et al. [23]. Suwal et al. [50] demonstrated that maintaining constant conductivity in all compartments was necessary for optimizing the recovery of valuable peptides from marine protein hydrolysates during electrodialysis, with a linear relationship observed between conductivity and peptide migration. Thibodeau et al. [52], for their part, demonstrated that increasing the KCl concentration in the feed solution enhanced the migration of cationic peptides toward the cation exchange recovery fraction. In addition, a consistent ionic strength reduces electrostatic interactions between peptides and the ultrafiltration membrane to facilitate their migration [50]. At pH 7.0, the PES membrane shows a negative zeta potential [28]. As a result, the PES membrane exerted a strong electrostatic attraction toward cationic peptides, enhancing their migration. Concerning anionic peptides, their migration was hindered by electrostatic repulsions between the negatively charged peptides and the similarly charged membrane surface [20].

#### 3.1.3. Global System Resistance and Relative Energy Consumption

The global system resistance (GSR) of EDUF system started at 7.87 ± 0.37 Ω and decreased until 4.50 ± 0.19 Ω after 3 h of the process. These resistance values are relatively low compared to other studies performing EDUF with constant current on different matrices. Indeed, Cournoyer et al. [4] observed an increase in GSR from approximately 10 to 16 Ω over time whereas Adaile-Perez et al. [20] and Damen et al. [19] reported decreases in GSR from about 14 to 12 Ω and from 15.2 to 9.1 Ω, respectively. The decrease in GSR of the present study can be attributed to the similar conductivity values in all compartments. When one compartment exhibited higher conductivity than the other, the compartment with lower conductivity limited the transport of electric charges, resulting in increased overall resistance [53]. Conversely, and as observed in the present study, when all compartments have equal conductivity, no single compartment imposed a limiting effect, and the total system resistance reached its minimum [54,55]. In addition, the increasing conductivity of the hydrolysate and recovery fractions throughout the process gradually reduced the overall resistance of the EDUF system, thereby promoting more efficient ionic migration.

Relative energy consumption (REC) over the 3 h process was 89.2 ± 3.4 Wh/g for the P^+^ fraction and 127.1 ± 3.7 Wh/g for the P^−^ fraction, resulting in an average total peptide relative energy consumption of 52.4 ± 0.7 Wh/g. This average REC value was relatively high compared with values reported in other studies using different matrices (39.76 ± 4.77 Wh/g for porcine cruor hydrolysate [4] and 31.27 ± 2.6 Wh/g for chicken by-product hydrolysate [20]). The high conductivity observed in all fractions may explain the higher REC. Indeed, given the higher electrophoretic mobility of minerals compared to peptides, they migrated more easily and before the peptides. As a result, more energy was required to enable peptide migration [56,57].

#### 3.1.4. Membrane Characterization

**Figure 4:**
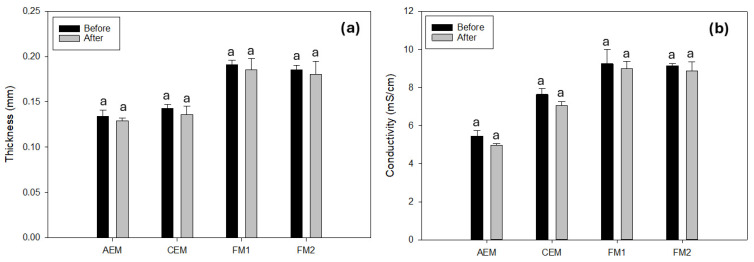
Membrane thickness (**a**) and electrical conductivity (**b**) values before and after EDUF process. Values are presented as the mean ± standard deviation. Similar letters indicate similar results based on paired *t*-tests (*p* > 0.05) before and after the EDUF process treatment for the same membrane. AEM: anion exchange membrane; CEM: cation exchange membrane; FM: filtration membrane.

All membranes used in the EDUF configuration were characterized for their thickness and conductivity before and after the separation process (Figure 4). The integrity of the membranes was not affected by EDUF since no statistical difference (*p* > 0.05) was observed. In addition, since the conductivities of the membranes remained similar, it can be concluded that no fouling phenomena occurred. A potential fouling would have reduced the availability of charge carriers on the membrane surface by the binding of ions from the CCH [58,59]. Anionic and cationic peptides which migrated toward the cathode and anode were then allowed to migrate through the membrane. Hence, there was a limited or no adsorption of the peptides on the membrane.

### 3.2. Hierarchical Clustered Heatmap of Calf Cruor Hydrolysate Fractions

**Figure 5:**
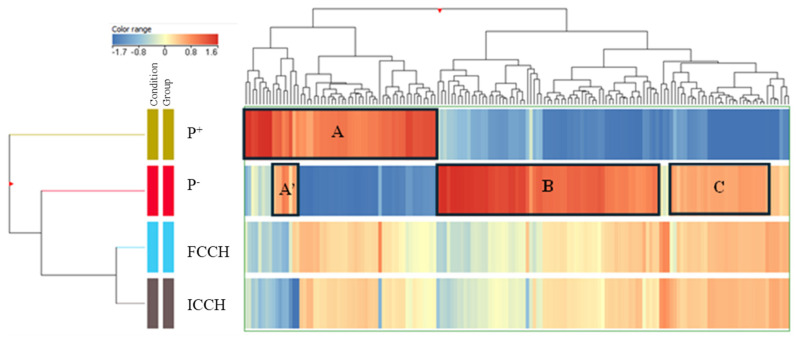
Hierarchical clustered heatmap of EDUF fractions from calf cruor hydrolysate. P^+^: cationic recovery compartment fraction; P^−^: anionic recovery compartment fraction; FCCH: final calf cruor hydrolysate; ICCH: initial calf cruor hydrolysate. Peptide similarities were calculated using Euclidean distance, and the resulting profiles are shown as a heatmap. The color gradient ranges from blue to red, indicating low (blue) to high (red) peptide intensities, reflecting the degree of up- or downregulation. The peptides within the same box (A, A’, B or C) having a short Euclidean distance showed similarities in their physico-chemical properties.

The hierarchical clustered heatmap obtained after statistical treatment of the different EDUF fractions showed, as expected, that the ICCH and FCCH had a very similar pattern as illustrated by the short Euclidean distance between the two fractions (Figure 5). Indeed, during EDUF treatment, the migration of peptides was very selective and the total quantity of migrated peptides, according to the chosen experimental conditions, was low due to the membrane surface and process duration as already reported in the literature (less than 16%) [60]. The ones that migrated are concentrated in either P^+^ or P^−^ recovery fraction during the three hours of EDUF treatment. Furthermore, the hierarchical clustered heatmap demonstrated that different groups of peptides migrated in the P^+^ and P^−^ recovery fractions identified by the red zones corresponding to the upregulated peptide sequences found in each one. Of the 157 peptide sequences identified and present in the heatmap, 56 have migrated from ICCH to P^+^ (Box A, with 8 common to P^−^ in Box A’) and 65 to P^−^ (Box B). Box C revealed 27 peptides that migrated to P^−^ and were slightly more abundant than in the ICCH and FCCH. A recent study by Damen et al. [19] obtained a similar heatmap with a white wastewater hydrolysate from the milk industry and EDUF fractions recovered after 3 h of process at 7.8 V.

To better understand the migration of peptides in the different boxes in Figure 5, a bioinformatic tool was used to determine different physico-chemical characteristics for these sequences as reported in Table S1. They were chosen according to previous studies on EDUF and peptide migration [4,27,28]: molecular weight (important for migration and according to the MWCO of the filtration membrane used), isoelectric point (pI; determines the charge of the peptides at working pH), GRAVY index (hydrophobicity of the peptides), and leucine (L, a hydrophobic amino acid, increasing the hydrophobicity of a peptide sequence [61]) and tyrosine (Y, its aromatic ring can impact binding to other peptides or membranes [29]) percentages in the sequences. To facilitate interpretation, Table 1 reports average values for the physico-chemical characteristics of the peptide sequences from Table S1 found within the upregulated boxes of the clustered heatmap (Figure 5) and provides a color-coded representation of trends.

When looking more closely at each box, and knowing that, in EDUF, the migration of peptides was influenced by the peptides physico-chemical characteristics as well as different types of interactions occurring between peptides and filtration membranes [28], it can be discussed of how physico-chemical characteristics of peptides and/or membranes intervene to help or hinder their migration. Hence, it appears that peptides in Box A (upregulated in P^+^), that migrated from ICCH, had high pI, molecular weight (MW), and %Y and the lowest GRAVY score and low %L (Table 1). This relatively high pI (average of 7.96; 39 sequences out of the 56 have a pI above 8; Table S1) provides them with positive charges at pH 7.0 and supports cation-directed migration towards P^+^ under the application of an electric field. These high peptides positive charges, which were the dominant features driving migration in the P^+^ fraction, also allowed for strong electrostatic attraction to the negatively charged PES membrane (zeta-potential at pH 7.0 of −13.4 mV [28]). In addition, their molecular weights (average of 867 Da and ranging between 327 and 2562 Da; Table S1), even if the highest of the different boxes, were still low MW. So, this ensured negligible steric hindrance given the membrane’s 50 kDa MWCO and 18% macropores and favored their migration [28,62]. The presence of high tyrosine percentage in some of the peptide sequences (18 out of 56 sequences; Table S1), such as AHRYH, KYR, SKYR, TSKY, or TSKYR, introduced an additional factor: π–π stacking interactions between tyrosine’s aromatic ring and the aromatic backbone of PES [63], which may promote adsorption at the membrane interface and reduce peptide migration [29]. The low GRAVY score (average of −0.21) and low leucine content (average of 13.1%) indicated a hydrophilic character for the peptides, which can lead to hydrophilic–hydrophilic interactions with the membrane’s highly hydrophilic surface (84% hydrophilic porosity and contact angle of 79° [28]), and may have slowed down the migration [64]. However, due to the high ionic strength throughout the process (Figure 2) these interactions were reduced [65] and since no measurable fouling was demonstrated, after membrane characterization (Figure 4), it indicated that migration was not affected. In addition, contrary to what could be expected, some negatively charged peptides were found in Box A. These exceptions can be explained by aggregate formation with oppositely charged peptides/ions by hydrophobic and/or electrostatic interactions: for instance, these three sequences in Box A; AETL, EYGAETL, and MSELSNL, had the lowest pI of 4.05 (Table S1) and were strongly negatively charged at pH 7.0, so they may bind positively charged peptides to yield a net positive complex [66], thus migrating in the P^+^ recovery fraction. Such bindings between peptides have already been reported and explained by Kadel et al. [28] and Geoffroy et al. [60].

Concerning Box A′ (P^−^; upregulated peptides common to Box A), eight sequences were present with the lowest average MW of all boxes, similar %Y as Box A and neutral pI/GRAVY/%L according to the color code in Table 1. These were downregulated in ICCH and FCCH and upregulated, thus, concentrated in P^+^ and P^−^ recovery fractions. An explanation as why they were found in both recovery fractions can be their pI (average of 6.25) and GRAVY score (average of −0.08) with values that suggests near-neutral charge and hydrophobicity, so they can exert mild electrostatic attraction to the negatively charged membrane when the electric field was applied favoring their migration in either recovery fraction. Also, their low MW (591 Da average but ranging from 327 to 781 Da (Table S1)) can favor migration since there was no steric hindrance, as described for Box A, to pass through the selected membrane [64]. Contrarily, their higher leucine content (average of 18.5%) than Box A may confer a slightly higher hydrophobic character to this box, especially for some sequences like EYGAETL, LSHSLL and PVL that, with their leucine side chains, can display hydrophobic areas and bind to other peptides and migrate even if the primary parameter that determined migration was the electric field [4]. Moreover, neutral peptides or some anionic peptides, that are present in ICCH, can migrate in both compartments by their charge (even if slightly negative at pH 7.0) and/or by forming aggregates with other anionic peptides, as described for Box A, migrating together in P^−^ [60]. Tyrosine content (4.9%) remained relevant, as π–π stacking interactions with PES could slightly hinder migration, but its influence was likely secondary compared to the hydrophobic contribution of leucine that would help peptides to migrate. Consequently, Box A’ peptides probably migrated aided by their small size and weaker overall charge.

Box B (P^−^; highly upregulated) combined low MW (compared to boxes A and C), pI, and %Y with high GRAVY and %L (Table 1). Consequently, because of the membrane’s high MWCO, the low MW peptides in Box B (average of 658 Da but ranging from 273 to 1649 Da (Table S1)) had no difficulty migrating from ICCH to P^−^ recovery fraction. Moreover, the low pI (average of 5.19) rendered them highly negatively charged at pH 7.0 and, even if they were subjected to electrostatic repulsion from the negatively charged PES membrane, the strong negative charge allowed them to migrate toward the anode when the electric field was applied. Furthermore, since tyrosine content was negligible (average of 0.8%) for peptide sequences in Box B, π–π stacking interactions were unlikely to play a role and slowed down their migration which was driven, as mentioned previously, by their highly negative charge. Their strong positive GRAVY score (average of 1.06) and high leucine content (average of 22.4%) indicated pronounced hydrophobicity, which minimized adsorption on the hydrophilic PES surface and may even favor migration [64]. The high leucine proportion was particularly important here (52 sequences out of the 65 found in this box contain leucine (Table S1)): while electrostatic repulsion hinders migration, leucine-driven hydrophobicity counteracted this effect by limiting peptide–membrane affinity, resulting in higher migration rates compared to other negatively charged peptides. Additionally, another membrane parameter that can have a positive correlation to anionic peptide migration was roughness Rz (average peak-to-valley) values. As described by Kadel et al. [28], since both the membrane and peptides are negatively charged, electrostatic repulsion occurs between them. But this repulsive energy barrier height decreased with an increase in roughness values of the membranes (9 µm for the PES 50 kDa in this study compared to an average of 7.7 µm for PES membranes MWCOs ranging from 5 to 300 kDa as reported by Kadel et al. [64]). This consequently had less of an impact on slowing down the migration of negatively charged peptides towards the P^−^ fraction under the effect of the electric field during EDUF, compared to the effect of smooth surface having the same degree of charge [28]. Therefore, for Box B, it can be concluded that, aside from tyrosine content, the other physico-chemical characteristics had a positive impact on the migration of peptide sequences in P^−^ fraction.

Finally, peptides in Box C had slightly higher normalized abundances (P^−^; upregulated) than those from ICCH and FCCH and exhibited peptides with slightly higher MW than Box B (average of 739 Da vs. 658 Da), and low pI, GRAVY, %L, and %Y (Table 1). As explained for previous boxes, the low MW of the peptides in ICCH did not hinder their migration when using this PES 50 kDa membrane. Furthermore, the peptides’ migration was favored by their low GRAVY score (average of −0.17) and low leucine content (average of 10.4%), indicating a hydrophilic nature, in combination with the high hydrophilic porosity of 84% and the contact angle of 79° of the membrane [28]. In addition, as explained for Box B, the membrane’s roughness (Rz), thus, lowered energy barrier height, may have helped these strongly negatively charged peptides to cross the membrane. Nevertheless, their lowest average pI value of 4.98 provided them with a strong negative charge at a working pH of 7.0, and the migration, under the applied electric field, would then be slowed down due to electrostatic repulsions from the PES membrane. Additionally, tyrosine content (2.1%) introduced a minor possibility of π–π stacking interactions, but this effect was likely overshadowed by strong electrostatic repulsions (only three sequences out of the 27 have tyrosine; AAEYG, AEYG and LDNIKNTY (Table S1)). Consequently, the low MW, GRAVY and %L helped these peptides to migrate from ICCH to P^−^ fraction, but globally, they had lower migration due to the strong electrostatic repulsions.

Altogether, these observations reinforced the view that peptide migration, from ICCH to both recovery fractions, during EDUF was governed by the interplay between MW, net charge (pI), hydrophobicity, specific residue content (L, Y), and membrane–peptide interactions.

### 3.3. Antimicrobial Activities

#### 3.3.1. Agar Well Diffusion

**Figure 6:**
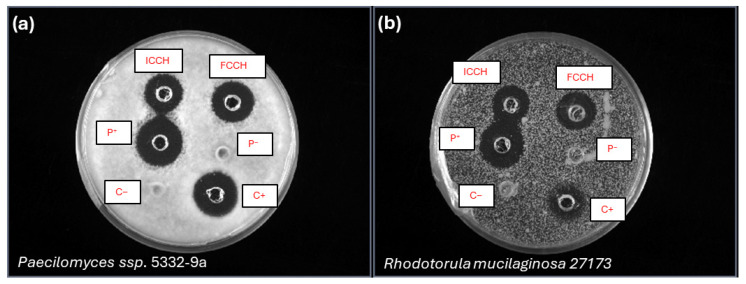
Agar well diffusion test results against (**a**) *Paecilomyces* spp. and (**b**) *Rhodotourula mucilaginosa*, with natamycin as the positive control (C+) and Milli-Q water as the negative control (C−) for initial and final hydrolysates (ICCH and FCCH) and recovery fractions (P^+^ and P^−^) after 180 min of the EDUF process.

Inhibition halos of the ICCH, FCCH and recovery fractions P^+^ and P^−^ were measured against fungal strains, revealing antifungal activity (Figure 6). No inhibition halos could be measured for *Escherichia coli* MP 4100 and *Listeria ivanovii* HP B28.

Inhibition halos were calculated for *Paecilomyces* spp. 5332-9a at 16 mm for the initial hydrolysate (ICCH), 17 mm for the final hydrolysate (FCCH), and 22 mm for P^+^ and for *R. mucilaginosa* 27,173 at 17 mm for the ICCH, 17 mm for the FCCH, and 20 mm for P^+^. No inhibition halo was observed for the solvent of hydrolysate fractions (Milli-Q water), which was used as the negative control (C−), and 18 mm and 15 mm halos were measured for natamycin (50 µg/mL), used as the positive control (C+) for *Paecilomyces* spp. 5332-9a and *R. mucilaginosa* 27173, respectively.

#### 3.3.2. MIC and MBC/MFC

Minimal inhibitory concentrations (MICs) for the fungal indicator strains as well as minimal fungicidal concentrations (MFCs) are represented in Table 2. The ratio MFC/MIC revealed a fungicidal activity of all active hydrolysate fractions.

Spoilage fungi represent a significant concern in calf-derived products, as they can compromise safety, nutritional quality, and shelf-life, ultimately leading to economic losses [67,68]. In addition, fungal contamination in animal feed poses risks not only to animal health and performance but also to food safety through the possible carryover of mycotoxins into the human food chain [69]. The demonstrated antifungal potential of ICCH, FCCH, and P^+^ hydrolysates highlight their potency as natural alternatives to conventional chemical preservatives. Their application in food and feed systems could reduce reliance on synthetic antimicrobials, aligning with current consumer demand for clean-label and sustainable solutions [70].

While ICCH, FCCH, and P^+^ hydrolysates exhibited strong antifungal activity, the P^−^ fraction did not show any antifungal effects. However, a slight anti-*Listeria* activity was detected, with a MIC of 10 mg/mL. No inhibition halos were observed against this strain, which may be attributed either to the relatively weak antimicrobial effect or to limited peptide diffusion within the agar matrix. Despite its modest activity, the anti-*Listeria* effect is noteworthy, as *Listeria* spp. represents major foodborne pathogens [40], posing significant risks to food safety. Further in-depth studies on the peptide composition of these hydrolysates are required to identify potential anti-listerial peptides with applications in the food industry.

Overall, the P^−^ compartment was characterized by low molecular weight peptides, low tyrosine (Y) content, and moderate to high leucine (L) content. These physico-chemical characteristics could help explain the slight anti-listerial activity observed. Low molecular weight peptides generally exhibited enhanced diffusion and membrane penetration capabilities, facilitating their interaction with and disruption of bacterial cellular components [71,72]. In addition, amino acid composition played a key role in the antimicrobial activity of peptides. Leucine, a hydrophobic amino acid, contributed to the amphipathic nature of peptides, whereas tyrosine, although partially hydrophobic, introduced polar features that may modulate peptide–membrane interactions [73]. When appropriately arranged, the segregation of hydrophobic and polar residues promoted amphipathic structures that facilitated membrane insertion and bacterial membrane disruption [74].

Peptide sequences present in each fraction were analyzed using bioinformatic tools, querying multiple databases to predict potential bioactivities. Table 3 summarizes the sequences identified in the P^+^ and P^−^ fractions that display potential bioactive properties. Each peptide exhibited a net charge at pH 7.0 corresponding to its migration compartment. Notably, the cationic fraction was enriched in sequences with already known antibacterial activities, as seven out of 56 identified sequences are known to possess antibacterial properties. These peptides were successfully concentrated in the P^+^ fraction following 3 h of EDUF.

Recent studies on calf cruor by Sanchez-Reinoso et al. [9,75] employed a machine learning approach to identify novel antimicrobial peptides. Several sequences were synthesized and tested against a broad spectrum of bacterial and fungal strains. In the present study, two peptide sequences (HAHKLRVDPVNF and QKVVAGVANALAHRYH), not previously reported in databases but present in the cationic fraction, were recently demonstrated to exhibit strong antifungal activity against three strains (*R. mucilaginosa* 27,173; *Paecilomyces* spp. 5332-9a; and *Mucor racemosus*). Their physico-chemical characteristics and MIC values are summarized in Table 4.

The ICCH, FCCH, and P^+^ fractions showed strong antifungal activity and therefore hold potential for application in the food industry, offering natural solutions to the growing demand for novel antimicrobials [76], which also include antifungal agents.

The case of P^−^ fraction, although the observed anti-listerial activity was associated with a relatively high MIC value, further investigations are needed to identify the specific peptides responsible for this effect within its complex peptide population. Antimicrobial peptides with anti-listerial properties are of particular interest to the food industry, as they may provide effective natural alternatives to conventional antimicrobials against *Listeria monocytogenes*, a major foodborne pathogen. This is especially relevant in the context of rising antimicrobial resistance [77], where the discovery of novel, naturally derived antimicrobial peptides could help alleviate the pressure on traditional antibiotics. Future research should therefore focus on the identification, characterization, and possible synthesis of the anti-*Listeria* peptides from the P^−^ fraction, paving the way for innovative biopreservative strategies that enhance food safety while addressing global sustainability challenges.

## 4. Conclusions

In this study, EDUF was performed, for the first time, for the fractionation of a calf cruor hydrolysate, a source of antimicrobial peptides, released through hydrolysis with pepsin. The EDUF process effectively separated a wide range of peptides, with high migration rates of 12.75 ± 2.17 g/m^2^h and 8.94 ± 0.38 g/m^2^h for the P^+^ and P^−^ fractions, respectively. This study on the separation of calf cruor hydrolysate by EDUF, a new source of bioactive peptides, successfully separated the peptides according to their size and charge, based on their physico-chemical characteristics. This study complements and further enhances the understanding of peptide migration based on their characteristics compared to previous works on porcine cruor peptides and bovine hemoglobin [4,23]. Indeed, these findings suggest that understanding peptide physico-chemical characteristics and their interactions with the membrane were critical for targeted peptide fractionation, especially in complex hydrolysates like calf cruor, where peptide diversity is high and functional bioactivity is desired. The optimization of the separation conditions (choice of working pH or membrane material to favor either anionic or cationic peptide enhanced fractions) could further improve peptide yield and enrich the fraction in bioactive sequences. ICCH and FCCH, as well as the P^+^ fraction, exhibited activity against *Paecilomyces* spp. and *R. mucilaginosa*. Furthermore, slight anti-*Listeria* activity was detected, with a MIC of 10 mg/mL. Although database analysis did not reveal any known antimicrobial peptides in the P^−^ fraction, three peptides with other bioactivities were identified. Hence, it would be of interest to further evaluate other bioactivities of this fraction, in addition to that of the P^+^ fraction, which also exhibits relevant bioactivities, particularly ACE-inhibitory activity. Overall, these findings emphasized the potential of bioactive peptides as innovative biopreservatives, providing natural alternatives to conventional antimicrobials and reinforcing global efforts toward sustainable food production and improved public health with a circular economic perspective.

## Figures and Tables

**Table 1:** Average values of each physico-chemical characteristic of the peptide sequences from Table S1 found in the different upregulated boxes of the clustered heatmap.

Box	Molecular Weight (Da)	Isoelectric Point	GRAVY	%L	%Y
A	867.06	7.96	−0.21	13.1	4.6
A’	591.29	6.25	−0.08	18.5	4.9
B	658.80	5.19	1.06	22.4	0.8
C	739.74	4.98	−0.17	10.4	2.1

GRAVY: grand average of hydropathy; %L: percentage of leucine; %Y: percentage of tyrosine. 
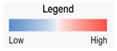
.

**Table 2:** MIC and MFC values (mg/mL) of the different hydrolysates tested against the fungal indicator strains.

	*Paecilomyces* spp. 5332-9a			*R. mucilaginosa* 27,173		
	MIC (mg/mL)	MFC (mg/mL)	MFC/MIC	MIC (mg/mL)	MFC (mg/mL)	MFC/MIC
ICCH	0.312 ± 0.000	0.312 ± 0.000	1	0.312 ± 0.000	0.615 ± 0.000	2
FCCH	0.312 ± 0.000	0.615 ± 0.000	2	0.312 ± 0.000	0.615 ± 0.000	2
P^+^	0.312 ± 0.000	0.615 ± 0.000	2	0.615 ± 0.000	1.250 ± 0.000	2
P^−^	>20	>20	ND	>20	>20	ND

ND: not determined.

**Table 3:** Peptide sequences with reported bioactivities found in anionic and cationic compartments after 3 h of EDUF with calf cruor hydrolysate.

Anionic Compartment						
Sequence	Molecular Weight (Da)	Isoelectric Point	GRAVY	Net Charge at pH 7	Potential Bioactivity	Database
LSF	365.430	5.525	1.933	−0.002	Multifunctional peptides	DFBP
VVL	329.441	5.495	4.067	−0.002	ACE inhibitor	BioPep
VVV	315.414	5.495	4.200	−0.002	Anticancer	BioPep
**Cationic compartment**						
FQKVVA	690.843	8.750	0.933	0.992	ACE inhibitor	BioPep
KLLSHSL	796.968	8.757	0.386	1.087	Anti-Gram+, Anti-Gram−	DBAASP
KLLSHSLL	910.128	8.757	0.812	1.087	Anti-Gram+, Anti-Gram−	DBAASP
KYR	465.555	9.994	−3.233	1.988	Anti-Gram+, Anti-Gram−	DBAASP
LAHRYH	795.903	8.762	−1.100	1.194	Anti-Gram+, Anti-Gram−	DBAASP
PHF	399.451	7.171	−0.667	0.098	Antioxidative	BioPep
PTTKTYFPHF	1238.411	9.007	−0.810	1.097	Anti-Gram+, Anti-Gram−	DBAASP
SKYR	552.633	9.992	−2.625	1.992	Anti-Gram+, Anti-Gram−	DBAASP
TSKYR	653.738	9.992	−2.240	1.991	Anti-Gram−, Anti-Gram+/ Opioid peptides	dbAMP/DFBP
VVYPWTQRF	1195.390	8.718	−0.144	0.997	Opioid, ACE inhibitor	BioPep

**Table 4:** Physico-chemical characteristics of two peptide sequences with antifungal activity from calf cruor hydrolysates [9,75].

Sequence	Molecular Weight (Da)	Isoelectric Point	GRAVY	Net Charge at pH 7	Bioactivity	MIC Value (mg/mL)
					*R. mucilaginosa* (27,173)	0.16 ± 0.00
HAHKLRVDPVNF	1432.654	8.762	−0.550	1.191	*M. racemosus*	1.25 ± 0.00
					*R. mucilaginosa* (27,173)	0.08 ± 0.00
					*Paecilomyces* spp. (5332-9a)	0.02 ± 0.00
QKVVAGVANALAHRYH	1734.001	9.994	0.006	2.189	*M. racemosus*	0.31 ± 0.00

## Data Availability

Data is contained within the article.

## Supplementary Materials

The following supporting information can be downloaded at: https://www.mdpi.com/article/10.3390/membranes16060202/s1. Table S1: Physico-chemical characteristics of the peptide sequences found in the different upregulated boxes of the clustered heatmap. Letters (A, A’, B, and C) correspond to boxes in Figure 5.
