# Supplementary material for: Assessment of Peptides and Membrane Physico-Chemical Characteristics on Migration Selectivity and Recovery of Antimicrobial Fractions Using Electrodialysis with Ultrafiltration Membrane on a Calf Cruor Hydrolysate
Source: Membranes (Basel). 2026 Jun 10;16(6):202. doi: 10.3390/membranes16060202 (PMC13303644; doi:10.3390/membranes16060202)
Supplement: Supplementary file 1 [file membranes-16-00202-s001.zip › membranes-4213677-supplementary.pdf]

Table S1. Physico-chemical characteristics of the peptide sequences found in the different upregulated boxes of the clustered heatmap. Letters (A, A’, B and C) correspond to boxes in Figure 5. GRAVY: Grand Average of Hydropathy; %L: Percentage of leucine; %Y: Percentage of tyrosine.

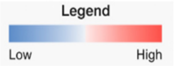

Calf Cruor Hydrolysate	P^+	P^-	Molecular Weight (Da)	Isoelectric Point	GRAVY	%L	%Y
AETL	A		432.474	4.05	0.350	0.250	0.000
AHRYH	A		682.743	8.80	-2.080	0.000	0.200
ARNFGKE	A		820.906	8.79	-1.600	0.000	0.000
ASHQEE	A	A'	699.675	4.81	-2.117	0.000	0.000
ASLDKF	A	A'	679.772	5.88	0.033	0.167	0.000
AVHMGKDYTPE	A		1247.397	5.51	-0.927	0.000	0.091
CGAEALARL	A		903.074	5.99	0.789	0.222	0.000
CQAA	A	A'	391.450	5.52	0.650	0.000	0.000
DLSHGSAQVKGHGAKVAAAL	A		1917.161	8.61	0.050	0.100	0.000
EEKQLITGLW	A		1216.402	4.53	-0.430	0.200	0.000
EYGAETL	A	A'	781.817	4.05	-0.543	0.143	0.143
FKL	A		406.527	8.75	0.900	0.333	0.000
FKLLG	A		576.739	8.75	1.220	0.400	0.000
FLSFPTTKTYFPH	A		1585.826	8.60	-0.177	0.077	0.077
FQKVVA	A		690.843	8.75	0.933	0.000	0.000
FQKVVAGVANA	A		1103.290	8.75	0.864	0.000	0.000
GKVGGHA	A		624.700	8.76	-0.329	0.000	0.000
HAHKLRVDPVNF	A		1432.654	8.76	-0.550	0.083	0.000
HGKKVLSSFGEAVKNLDNIKNTY	A		2562.915	9.40	-0.678	0.087	0.043
KAAVTAF	A		706.842	8.75	1.114	0.000	0.000
KGTF	A		451.524	8.75	-0.550	0.000	0.000
KLLGNVL	A		755.959	8.75	1.114	0.429	0.000
KLLSHS	A	A'	683.808	8.76	-0.183	0.333	0.000
KLLSHSL	A		796.968	8.76	0.386	0.429	0.000
KLLSHSLL	A		910.128	8.76	0.813	0.500	0.000
KYR	A		465.555	9.99	-3.233	0.000	0.333
LAHRYH	A		795.903	8.76	-1.100	0.167	0.167
LGR	A		344.416	9.75	-0.367	0.333	0.000
LGRL	A		457.576	9.75	0.675	0.500	0.000
LKGTF	A		564.684	8.75	0.320	0.200	0.000

LSFPTTKT	A		894.037	8.75	-0.225	0.125	0.000
LSFPTTKTYF	A		1204.390	8.59	-0.030	0.100	0.100
LSHSLL	A	A'	668.793	6.74	1.100	0.500	0.000
LVVYPWTQRF	A		1308.550	8.75	0.250	0.100	0.100
MSELSNL	A		792.910	4.05	0.129	0.286	0.000
PHF	A		399.451	7.17	-0.667	0.000	0.000
PTTKTYFPHF	A		1238.411	9.01	-0.810	0.000	0.100
PVL	A	A'	327.425	5.95	2.133	0.333	0.000
QKVVAGVA	A		770.930	8.75	1.050	0.000	0.000
QKVVAGVANA	A		956.113	8.75	0.670	0.000	0.000
QKVVAGVANAL	A		1069.273	8.75	0.955	0.091	0.000
QKVVAGVANALAHRYH	A		1734.001	9.99	0.006	0.063	0.063
QRF	A		449.512	9.75	-1.733	0.000	0.000
SAADKGNV	A		760.803	5.55	-0.538	0.000	0.000
SDLHA	A		541.562	5.06	-0.380	0.200	0.000
SHS	A		329.313	6.46	-1.600	0.000	0.000
SKYR	A		552.633	9.99	-2.625	0.000	0.250
TPEVHASLDKFLCA	A		1530.769	5.29	0.250	0.143	0.000
TQRF	A		550.617	9.41	-1.475	0.000	0.000
TSKY	A	A'	497.549	8.26	-1.675	0.000	0.250
TSKYR	A		653.738	9.99	-2.240	0.000	0.200
TVLTSKYR	A		967.136	9.99	-0.488	0.125	0.125
VAGVANALAHRYH	A		1378.562	8.73	0.254	0.077	0.077
VLTSKYR	A		866.031	9.99	-0.457	0.143	0.143
VVVAIHHPAALT	A		1227.477	6.89	1.467	0.083	0.000
VVYPWTQRF	A		1195.390	8.72	-0.144	0.000	0.111
AAEYG		C	509.516	4.05	-0.320	0.000	0.200
AEYG		C	438.437	4.05	-0.850	0.000	0.250
ESFGDL		C	666.685	4.05	-0.267	0.167	0.000
FES		C	381.385	4.05	-0.500	0.000	0.000
FLV		C	377.485	5.53	3.600	0.333	0.000
GDL		C	303.315	4.05	-0.033	0.333	0.000
GDLSTAD		C	677.665	4.05	-0.471	0.143	0.000
HVDPEN		C	709.714	4.35	-1.850	0.000	0.000
HVDPENF		C	856.891	4.35	-1.186	0.000	0.000
LANVSTVL		C	815.967	5.53	1.600	0.250	0.000

LDNIKNTY		C	980.087	5.83	-1.013	0.125	0.125
LGNI		C	415.491	5.53	1.100	0.250	0.000
LTAEE		C	561.589	4.24	-0.420	0.200	0.000
MLTAE		C	563.674	4.60	0.660	0.200	0.000
MLTAEE		C	692.789	4.24	-0.033	0.167	0.000
MLTAEEKA		C	892.043	4.53	-0.288	0.125	0.000
MLTAEEKAAVT		C	1163.360	4.53	0.273	0.091	0.000
MLTAEEKAAVTAF		C	1381.616	4.53	0.585	0.077	0.000
RNFGK		C	620.712	11.00	-1.900	0.000	0.000
SQCIQ		C	577.661	5.24	-0.160	0.000	0.000
SSFGEAVKNLD		C	1166.254	4.67	-0.345	0.091	0.000
TAEE		C	448.429	4.24	-1.475	0.000	0.000
TAEEKAAVT		C	919.000	4.53	-0.300	0.000	0.000
TAF		C	337.376	5.18	1.300	0.000	0.000
TKAVEHLDDLPGALSE		C	1694.861	4.53	-0.338	0.188	0.000
TSK		C	334.373	8.41	-1.800	0.000	0.000
WGKVKVDEVGGEAL		C	1486.691	4.68	-0.157	0.071	0.000
AAL		B	273.333	5.57	2.467	0.333	0.000
AALSEL		B	602.686	4.05	1.150	0.333	0.000
AEEKAAVTAF		B	1036.151	4.53	0.260	0.000	0.000
AEY		B	381.385	4.05	-1.000	0.000	0.333
ALTKAVEHLDDLPGAL		B	1662.907	4.54	0.281	0.250	0.000
ANVSTVL		B	702.807	5.57	1.286	0.143	0.000
ASHLPSD		B	725.757	5.34	-0.614	0.143	0.000
ASHLPSDFTPAVH		B	1378.510	5.97	-0.077	0.077	0.000
ASHLPSDFTPAVHASL		B	1649.827	5.97	0.238	0.125	0.000
AVTAF		B	507.588	5.57	1.980	0.000	0.000
DDLPGAL		B	699.759	4.05	0.057	0.286	0.000
DKLHVDPEN		B	1066.137	4.54	-1.633	0.111	0.000
DLSHGSA		B	685.692	5.08	-0.443	0.143	0.000
DLSHGSAQ		B	813.823	5.08	-0.825	0.125	0.000
EEKAAVTAF		B	965.072	4.53	0.089	0.000	0.000
EKAAVTAF		B	835.957	6.10	0.538	0.000	0.000
FESF		B	528.562	4.05	0.325	0.000	0.000
FESFGDL		B	813.862	4.05	0.171	0.143	0.000
FLA		B	349.431	5.53	2.800	0.333	0.000

FLSF		B	512.607	5.53	2.150	0.250	0.000
FTPVL		B	575.707	5.53	1.700	0.200	0.000
GAEAL		B	459.500	4.05	0.700	0.200	0.000
HLDDLPGAL		B	950.061	4.20	0.111	0.333	0.000
IVL		B	343.468	5.53	4.167	0.333	0.000
KDF		B	408.455	5.84	-1.533	0.000	0.000
LANV		B	415.491	5.53	1.575	0.250	0.000
LANVS		B	502.569	5.53	1.100	0.200	0.000
LGNVL		B	514.624	5.53	1.580	0.400	0.000
LLI		B	357.495	5.53	4.033	0.667	0.000
LLS		B	331.413	5.53	2.267	0.667	0.000
LLV		B	343.468	5.53	3.933	0.667	0.000
LLVT		B	444.573	5.53	2.775	0.500	0.000
LLVV		B	442.601	5.53	4.000	0.500	0.000
LSAADK		B	603.674	5.84	-0.133	0.167	0.000
LSAV		B	388.465	5.53	2.250	0.250	0.000
LSE		B	347.368	4.60	-0.167	0.333	0.000
LSEL		B	460.528	4.05	0.825	0.500	0.000
LSELSD		B	662.694	4.05	-0.167	0.333	0.000
LSF		B	365.430	5.53	1.933	0.333	0.000
LTAEEKA		B	760.843	4.53	-0.600	0.143	0.000
LTKAVE		B	659.782	6.22	0.283	0.167	0.000
LTKAVEH		B	796.924	6.75	-0.214	0.143	0.000
LTKAVEHLDDLPGAL		B	1591.828	4.54	0.180	0.267	0.000
LTPEVH		B	694.787	5.24	-0.167	0.167	0.000
LTRMF		B	666.846	9.75	0.660	0.200	0.000
LVTL		B	444.573	5.53	2.775	0.500	0.000
LVVVL		B	541.734	5.53	4.040	0.400	0.000
MLT		B	363.480	5.28	1.667	0.333	0.000
NLSQAM		B	662.767	5.53	-0.050	0.167	0.000
QADF		B	479.490	4.05	-0.600	0.000	0.000
STVL		B	418.491	5.24	1.625	0.250	0.000
TAEEKA		B	647.683	4.53	-1.333	0.000	0.000
TAEEKAAVTAF		B	1137.256	4.53	0.173	0.000	0.000
TKAVEHLDDLPGAL		B	1478.668	4.53	-0.079	0.214	0.000
VEHLDDLPGAL		B	1178.309	4.05	0.155	0.273	0.000

VGGEALGRL			B	871.007	5.97	0.489	0.222	0.000
VHWSAE			B	727.776	5.47	-0.400	0.000	0.000
VLQAD			B	544.606	4.30	0.560	0.200	0.000
VNVVA			B	500.597	5.49	2.180	0.000	0.000
VSTVL			B	517.624	5.49	2.140	0.200	0.000
VTL			B	331.413	5.49	2.433	0.333	0.000
VVL			B	329.441	5.49	4.067	0.333	0.000
VVV			B	315.414	5.49	4.200	0.000	0.000
VVVL			B	428.574	5.49	4.100	0.250	0.000
YGAEAL			B	622.676	4.05	0.367	0.167	0.167
